# Effectiveness of long-term graduated compression stockings after deep vein thrombosis: a GRADE-based meta-analysis of randomized controlled trials

**DOI:** 10.3389/fmed.2026.1840243

**Published:** 2026-05-21

**Authors:** Yuanjie Duan, Juan Liu, Shiyu Lin, Xi Li, Dan Luo, Lemei Zhu

**Affiliations:** 1 Hunan Key Laboratory of the Research and Development of Novel Pharmaceutical Preparations, School of Public Health, Changsha Medical University, Changsha, China; 2 Hunan Provincial University Key Laboratory of the Fundamental and Clinical Research on Neurodegenerative Diseases, Changsha Medical University, Changsha, China; 3 School of Public Health, Changsha Medical University, Changsha, China

**Keywords:** deep venous thrombosis, elastic compression stockings, meta-analysis, post-thrombotic syndrome, prophylaxis

## Abstract

**Background:**

Post-thrombotic syndrome (PTS) is a common long-term complication following deep vein thrombosis (DVT), leading to substantial morbidity and impaired quality of life. Elastic compression stockings (ECS) are widely used for PTS prevention; however, their effectiveness remains controversial, particularly across different severities of PTS.

**Method:**

A systematic review and meta-analysis of randomized controlled trials (RCTs) was conducted to evaluate the effectiveness of ECS in preventing PTS after DVT. Electronic databases were searched from inception to January 20, 2026. The primary outcomes were the overall incidence of PTS at final follow-up, as well as the incidence of mild-to-moderate and severe PTS. Secondary outcomes included recurrent DVT and all-cause mortality. Risk ratios (RRs) with 95% confidence intervals (CIs) were pooled using a random-effects model. The certainty of evidence was assessed using the GRADE approach.

**Results:**

Eight trials involving 1,775 patients were included. ECS were associated with a modest reduction in total PTS (RR 0.70; 95% CI 0.51–0.96; absolute reduction ~127 per 1,000), corresponding to an absolute reduction of approximately 142 cases per 1,000 patients. When stratified by severity, ECS reduced mild-to-moderate PTS (RR 0.69; 95% CI 0.50–0.93; absolute reduction ~129 per 1,000), whereas effects on severe PTS were uncertain (RR 0.56; 95% CI 0.22–1.43). No clear impact was observed on recurrent DVT (RR 0.90; 95% CI 0.73–1.11) or all-cause mortality (RR 0.98; 95% CI 0.68–1.40). Certainty of evidence was low for total and mild-to-moderate PTS and very low for severe PTS, recurrent DVT, and mortality. Heterogeneity in adherence, control group design, PTS definitions, and initial DVT management may have influenced outcomes.

**Conclusion:**

Long-term ECS may modestly reduce mild-to-moderate PTS after DVT, but their effect on severe PTS, recurrent DVT, and mortality remains uncertain. ECS may improve symptoms, but their preventive effect is limited, and clinical decisions should consider patient-specific factors, adherence, and the multifactorial determinants of PTS.

**Systematic review registration:**

https://www.crd.york.ac.uk/PROSPERO/view/CRD420261386069.

## Introduction

Deep vein thrombosis (DVT) is a common vascular condition associated with substantial morbidity and long-term complications (1, 2). Among these, post-thrombotic syndrome (PTS) represents one of the most frequent and clinically significant sequelae, affecting up to 20%–50% of patients after DVT (3, 4). PTS is characterized by chronic pain, edema, skin changes, and in severe cases, venous ulceration, leading to impaired quality of life and increased healthcare burden (5). Given the lack of curative treatment, prevention of PTS remains a key therapeutic goal following DVT (6).

Elastic compression stockings (ECS) have long been recommended as a non-invasive intervention to improve venous return, reduce venous hypertension, and alleviate symptoms after DVT (6–8). However, the effectiveness of ECS in preventing PTS remains controversial. While earlier randomized trials and meta-analyses suggested a protective effect (9–11), more recent high-quality studies, such as the SOX trial, reported no significant benefit (12–14). Furthermore, most previous studies have evaluated PTS as a single outcome, without adequately distinguishing between different severities of the condition. This is clinically important, as mild-to-moderate PTS and severe PTS differ substantially in terms of pathophysiology, prognosis, and clinical impact (3, 5, 15).

In addition, the certainty of evidence regarding ECS remains unclear, partly due to heterogeneity in study design, variations in compression protocols, and inconsistent reporting of adherence (9, 16, 17). Therefore, a comprehensive evaluation incorporating both severity stratification and evidence certainty is warranted. The present study aimed to systematically assess the effectiveness of ECS in preventing PTS after DVT, with a particular focus on stratified outcomes according to disease severity and an evaluation of the certainty of evidence using the GRADE approach.

## Method

This meta-analysis was conducted and reported following the PRISMA (Preferred Reporting Items for Systematic Reviews and Meta-Analyses) guidelines (18). The study protocol was prospectively registered on PROSPERO (CRD420261386069).

### Search strategy and selection process

On January 20, 2026, a systematic search of Medline, Cochrane Library, Embase, and Web of Science was conducted to identify eligible studies from database inception to the search date. Detailed search strategies are provided in the Supplementary material S1. Study selection was independently performed by two reviewers. After removing duplicates, titles and abstracts were screened, and full texts of potentially eligible studies were retrieved for further assessment. The inclusion criteria were as follows: (1) patients with deep vein thrombosis; (2) the intervention group received elastic compression stockings; (3) the control group received placebo stockings without compression or no stockings; (4) stockings were worn for more than 1 year; (5) reported outcomes on post-thrombotic syndrome after 1 year of use. Exclusion criteria were: (1) non-randomized controlled trials; (2) full text not available; (3) non-English language studies. Any disagreements were resolved through discussion.

### Data extraction

Data were independently extracted from the included studies by two reviewers using a standardized data collection form. Extracted information encompassed study characteristics (including authors, publication year, study location, and registration details), participant demographics (sample size, age, and sex distribution), details of the intervention and control protocols, as well as follow-up duration. For studies that reported post-thrombotic syndrome (PTS), any available information on severity—such as mild-to-moderate or severe—was also recorded. Discrepancies were resolved through discussion. For studies with missing information, corresponding authors were contacted to obtain the required data.

### Risk of bias

Two independent reviewers assessed the risk of bias for all included randomized controlled trials (RCTs). The revised Cochrane Risk of Bias tool (RoB 2) was employed to evaluate potential biases across five key domains: (1) randomization process, including sequence generation and allocation concealment; (2) deviations from intended interventions, addressing whether participants received the planned interventions and the likelihood of bias affecting outcomes; (3) missing outcome data, evaluating the completeness of reported data and the potential impact of any omissions; (4) outcome measurement, examining blinding of assessors and the appropriateness of measurement methods; and (5) selection of reported results, assessing the risk of selective reporting (19). Each domain was classified as low risk, some concerns, or high risk based on the available evidence. An overall risk of bias for each study was derived by integrating the assessments across all five domains. Any disagreements between reviewers within a domain were resolved through discussion until consensus was reached.

### Outcome

In this meta-analysis, “long-term” was defined as ≥12 months of elastic compression stocking (ECS) use. Although the exact duration and follow-up varied across studies (12–24 months), all included trials assessed outcomes after at least 1 year of ECS use. The primary outcomes were the overall incidence of post-thrombotic syndrome (PTS) at the final follow-up after wearing elastic compression stockings, as well as the incidence of mild-to-moderate and severe PTS. PTS was diagnosed based on the Villalta scale, Ginsberg criteria, and CEAP classification (5). The definitions of mild-to-moderate and severe PTS were determined by each included study. The secondary outcomes included the incidence of recurrent lower-limb deep vein thrombosis (DVT) and all-cause mortality at the final follow-up after wearing the stockings.

### Statistical analysis

All outcomes, being binary variables, were summarized using risk ratios (RRs) with 95% confidence intervals (CIs). Pooled analyses were conducted using the Mantel–Haenszel random-effects model, and 95% CIs were calculated using the Hartung–Knapp–Sidik–Jonkman method (20, 21). Between-study heterogeneity was estimated by τ^2^ using the Restricted Maximum Likelihood method. The robustness of each outcome was assessed by a leave-one-out sensitivity analysis; an outcome was considered robust if the pooled effect remained stable after the removal of any single study, and non-robust if a substantial change occurred. Publication bias was evaluated using funnel plots and Begg’s test (22). We pre-specified subgroup analyses based on PTS severity (mild-to-moderate and severe), and also stratified by the control group intervention (placebo vs. no stocking). Additionally, considering the potential influence of adherence on the effect of compression stockings, we performed a meta-regression analysis based on adherence. When 10 or more studies were included, both methods were applied together to assess potential bias; when 10 or fewer studies were included, only Begg’s test was used.

### Quality of evidence

The GRADE approach was used to assess the quality of evidence for each outcome (23–29). Since all included studies were randomized controlled trials (RCTs), the following five domains were evaluated: risk of bias, imprecision, inconsistency, indirectness, and publication bias. In the absence of published, outcome-specific minimal important differences (MIDs) for binary outcomes, we adopted the “default” MID thresholds of risk ratios 0.8 and 1.25 to define clinically important benefit or harm, consistent with recent studies’ guidance when no established MID values are available (30, 31). For outcomes where the 95% CI did not cross the decision threshold, evidence was generally not downgraded; outcomes with <400 participants (continuous) or <300 events (binary) were downgraded one level for imprecision, and outcomes with CIs crossing the threshold were downgraded due to inconclusive effect (32).

## Result

### Study selection

**Figure 1:**
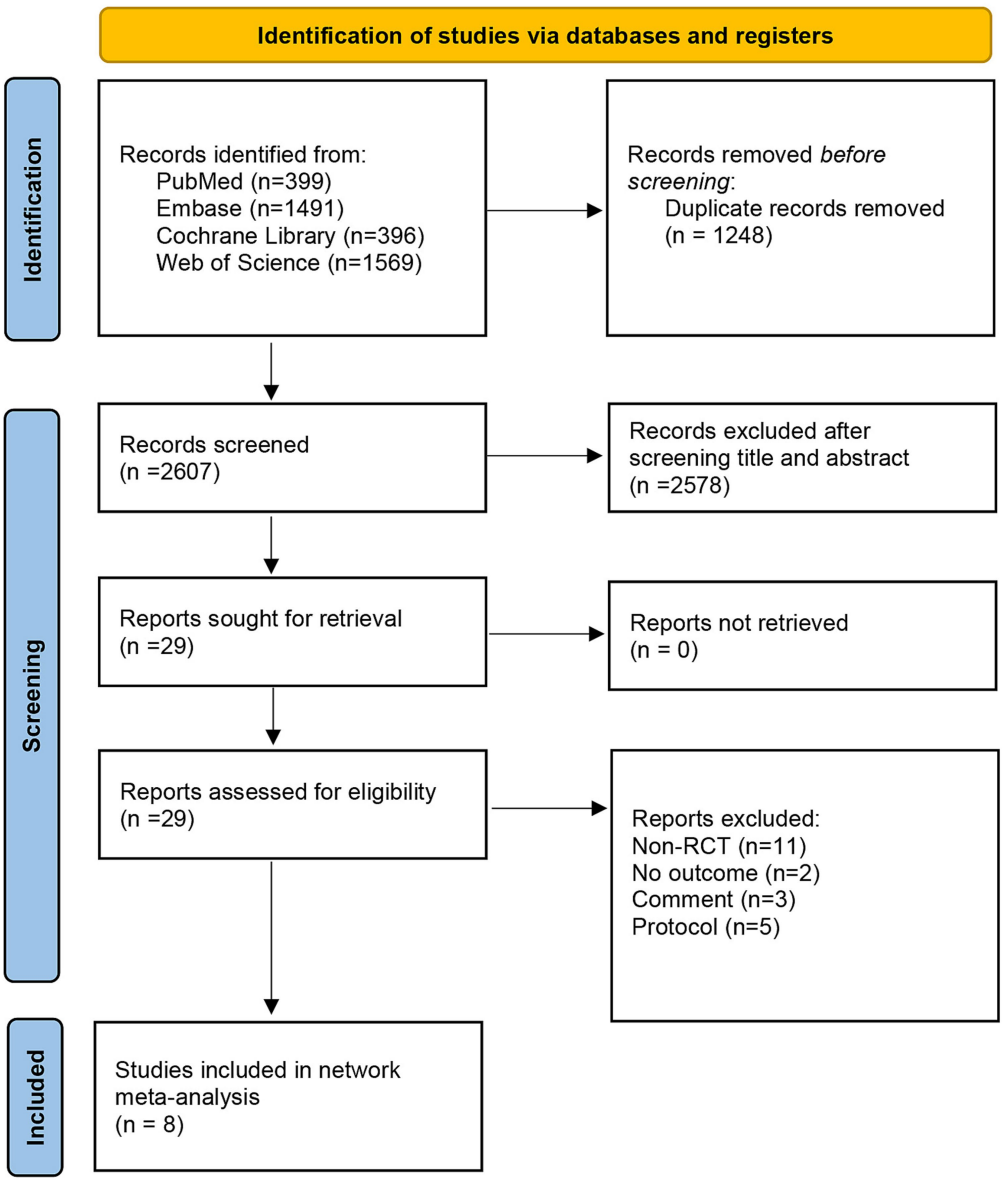
Flowchart of study selection.

A total of 3,855 records were identified through the literature search. After removing 1,248 duplicates, 2,607 records remained for title and abstract screening, of which 29 were selected for full-text review. Ultimately, eight studies meeting the inclusion and exclusion criteria were included in the analysis (Figure 1).

### Study characteristic and risk of bias

A total of eight studies including 1,775 patients were analyzed, with 895 patients in the intervention group and 880 in the control group (10, 12, 33–38). All included trials systematically excluded patients with pre-existing venous ulcers or advanced chronic venous disease at baseline, ensuring that the PTS assessment was not confounded by pre-existing ulceration. The mean age of participants ranged from 48 to 64 years in the intervention group and from 47 to 64 years in the control group. In these studies, the ankle pressure of elastic compression stockings ranged from 20 to 40 mmHg. Most included trials used either below-knee or knee-high elastic compression stockings, both primarily targeting the calf region; their minor differences are unlikely to meaningfully affect clinical effect. Two studies (12, 34) used placebo stockings, whereas in six studies (10, 33, 35–38) the control group did not wear any stockings. High adherence to stockings (>80%) was reported in three studies (10, 33, 36), good adherence (>60%) in four studies (12, 35, 37, 38), and one study (34) did not report adherence. Regarding PTS diagnosis, six studies (10, 12, 35–38) used the Villalta scale, two (12, 34) used the Ginsberg scale, and one (33) used the CEAP classification (Table 1).

**Table 1:** Study characteristic.

Study	Location	Diagnostic criteria for PTS	Male/Female		Mean age		Details of stockings		Compliance
			Intervention	Control	Intervention	Control	Intervention	Control	
Brandjes et al. (10)	Netherlands	Villalta	54/42	55/44	60	59	Knee high; Elastic progressive compression stocking group (ankle pressure 40 mmHg, lower calf 36 mmHg, upper calf 21 mmHg)	No stockings	93%
Ginsberg et al. (34)	Canada	Ginsberg	14/10	12/11	62	61	Knee high; Below-knee progressive compression stockings (20–30 mmHg)	Placebo stockings	NA
Prandoni et al. (36)	Italy	Villalta	42/48	35/55	60	63	Below knee; Ankle pressure 30–40 mmHg	No stockings	87%
Aschwanden et al. (33)	Switzerland	CEAP	54/30	45/40	64	64	Below-knee stockings with an ankle pressure ranging from 26.3 to 36.1 mmHg	No stockings	92%
Kahn et al. (12)	Canada	Villata/Ginsberg	255/145	228/166	55	55	Knee high; Graduated elastic compression stockings applying 30–40 mmHg pressure	Placebo stockings	69%
Jayaraj et al. (35)	America	Villalta	18/18	17/16	48	47	Below knee; Graduated compression stockings with 30–40 mmHg pressure	No stockings	60%
Yang et al. (38)	China	Villalta	48/65	52/67	61	58	Knee high; Knee-length elastic stockings with a daytime wearing pressure of 30–40 mmHg	No stockings	75%
Thapar et al. (37)	United Kingdom	Villalta	56/20	47/29	59	56	Below knee; Elastic compression stockings providing 23–32 mmHg pressure at the ankle	No stockings	60%

CEAP, clinical-etiology-anatomy-pathophysiology; PTS, post-thrombotic syndrome; NA, not available.

Among these eight studies, four (10, 12, 34, 38) were assessed as having low risk of bias. Of the remaining four studies, two (33, 36) were judged as high risk due to deviations from intended interventions, and two (35, 37) were judged as high risk due to a loss to follow-up exceeding 20% (Supplementary material S2).

### Post-thrombotic syndrome incidence

**Figure 2:**
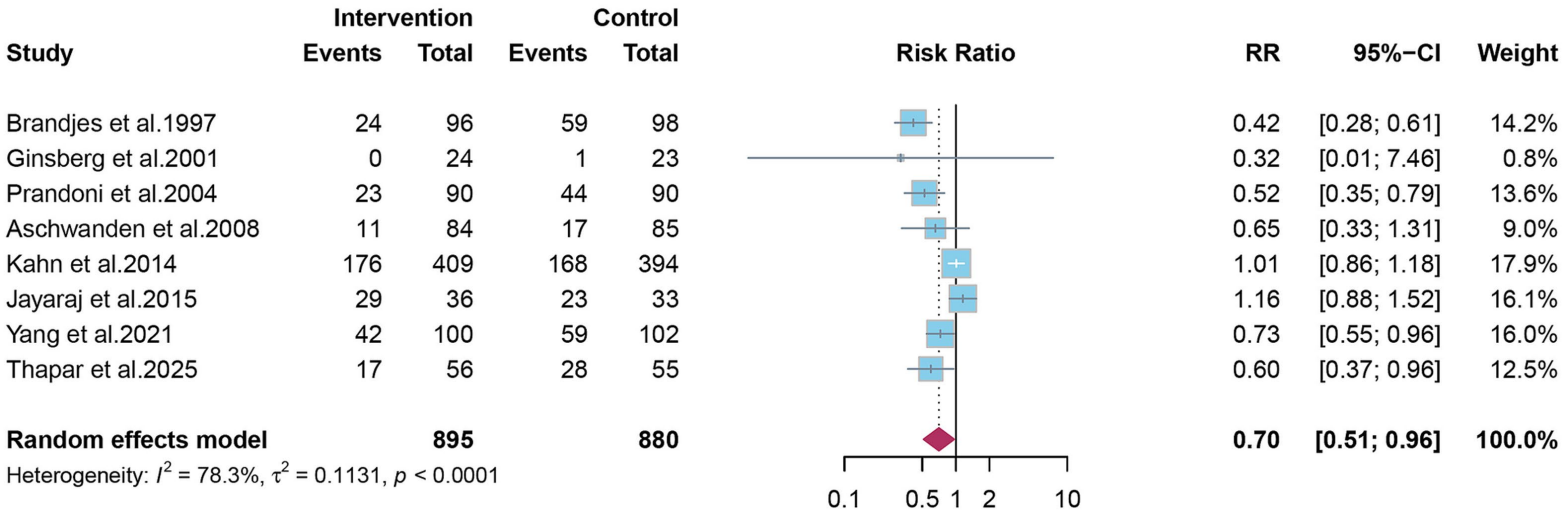
The effect of elastic compression stockings on overall incidence of post-thrombotic syndrome.

**Figure 3:**
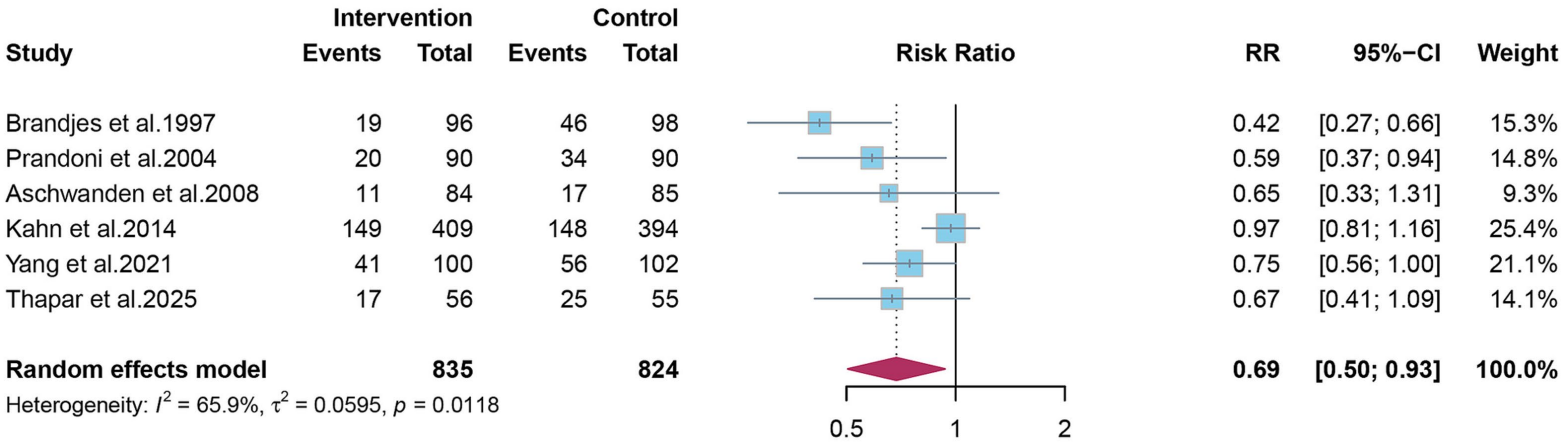
The effect of elastic compression stockings on incidence of mild-to-moderate post-thrombotic syndrome.

**Figure 4:**
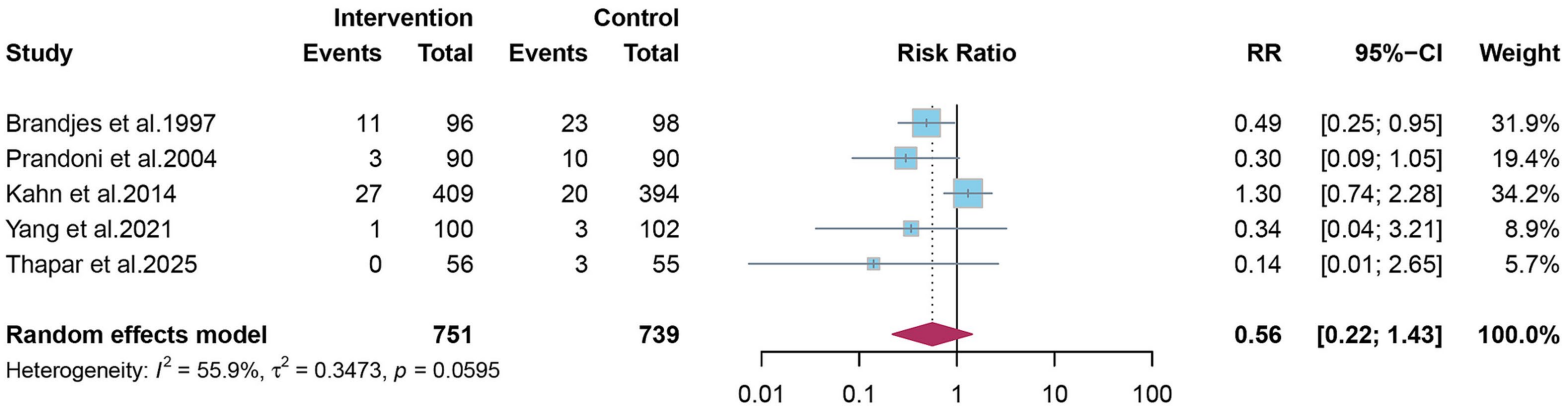
The effect of elastic compression stockings on incidence of severe post-thrombotic syndrome.

Eight studies reported the overall incidence of PTS. Pooled analysis showed that elastic compression stockings were associated with a 30% reduction in the overall incidence of PTS compared with placebo or no stockings (RR 0.70; 95% CI 0.51–0.96) (Figure 2). Given the clinical distinction between mild-to-moderate and severe PTS, subgroup analyses were conducted. Elastic compression stockings significantly reduced the incidence of mild-to-moderate PTS by 31% (RR 0.69; 95% CI 0.50–0.93) (Figure 3), whereas their effect on severe PTS remained uncertain (RR 0.56; 95% CI 0.22–1.43) (Figure 4).

### Recurrence of DVT and all-cause mortality

**Figure 5:**
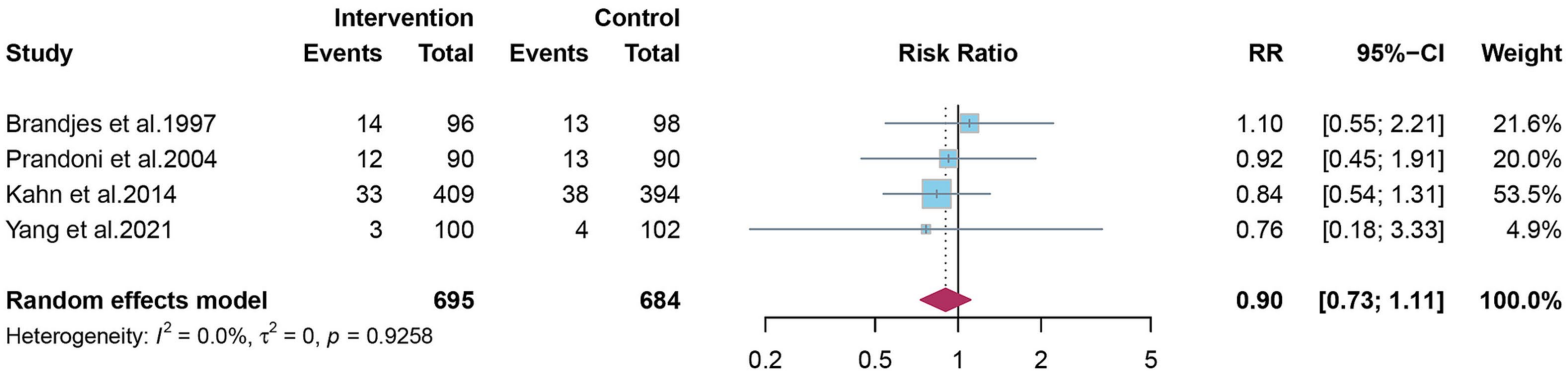
The effect of elastic compression stockings on incidence of DVT recurrence.

**Figure 6:**
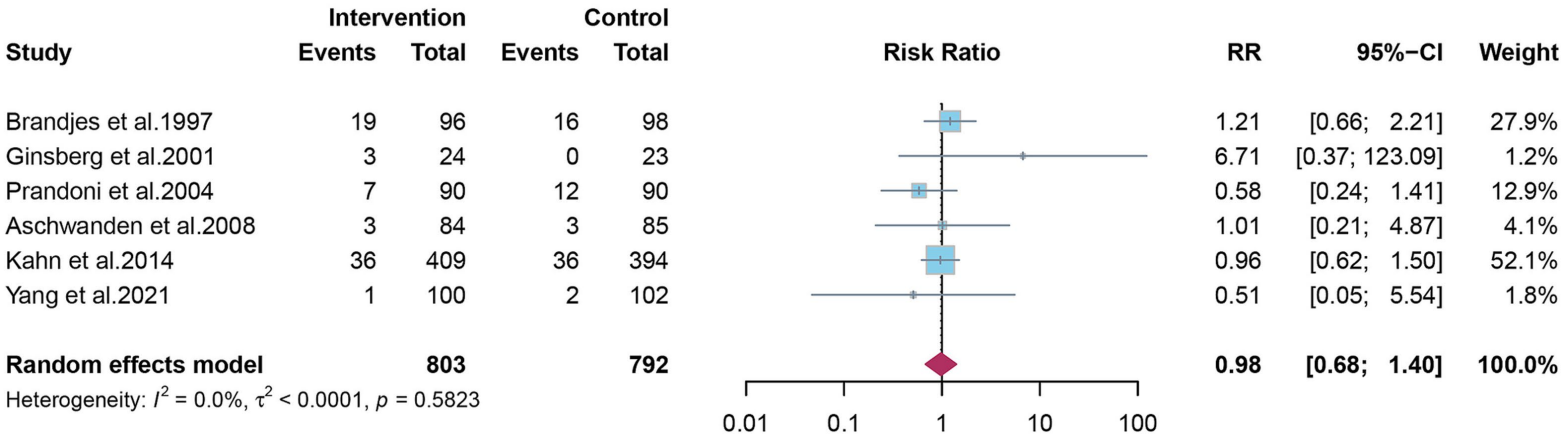
The effect of elastic compression stockings on incidence of all-cause mortality.

Four studies reported ipsilateral DVT recurrence. The pooled estimate indicated no clear preventive effect of elastic compression stockings (RR 0.90; 95% CI 0.73–1.11) (Figure 5). Six studies reported all-cause mortality during follow-up, with no significant difference observed between groups (RR 0.98; 95% CI 0.68–1.40) (Figure 6).

### Subgroup analysis, meta-regression, sensitivity analysis, publication bias, and certainty of evidence

**Figure 7:**
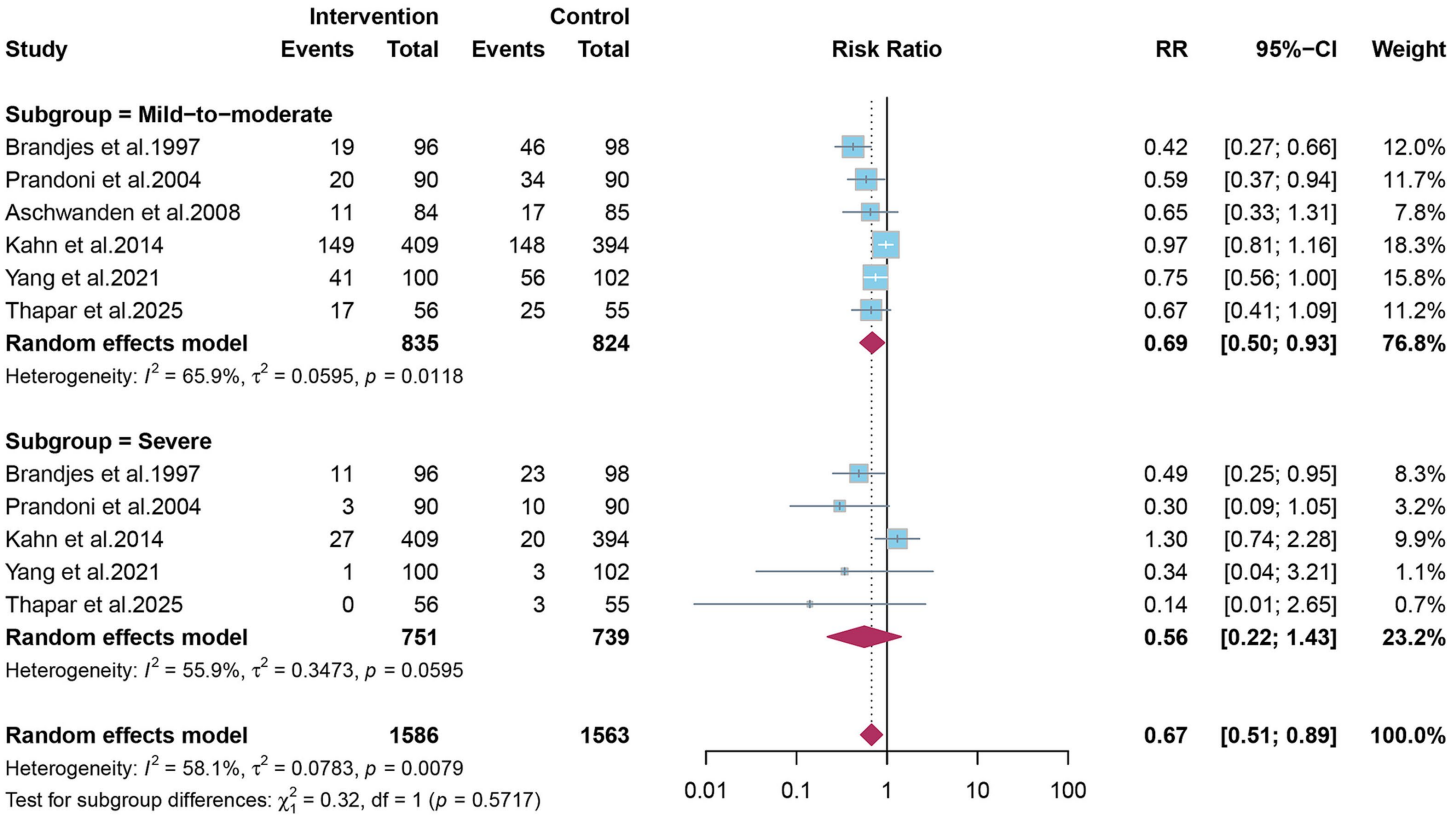
Subgroup analysis on PTS severity.

**Figure 8:**
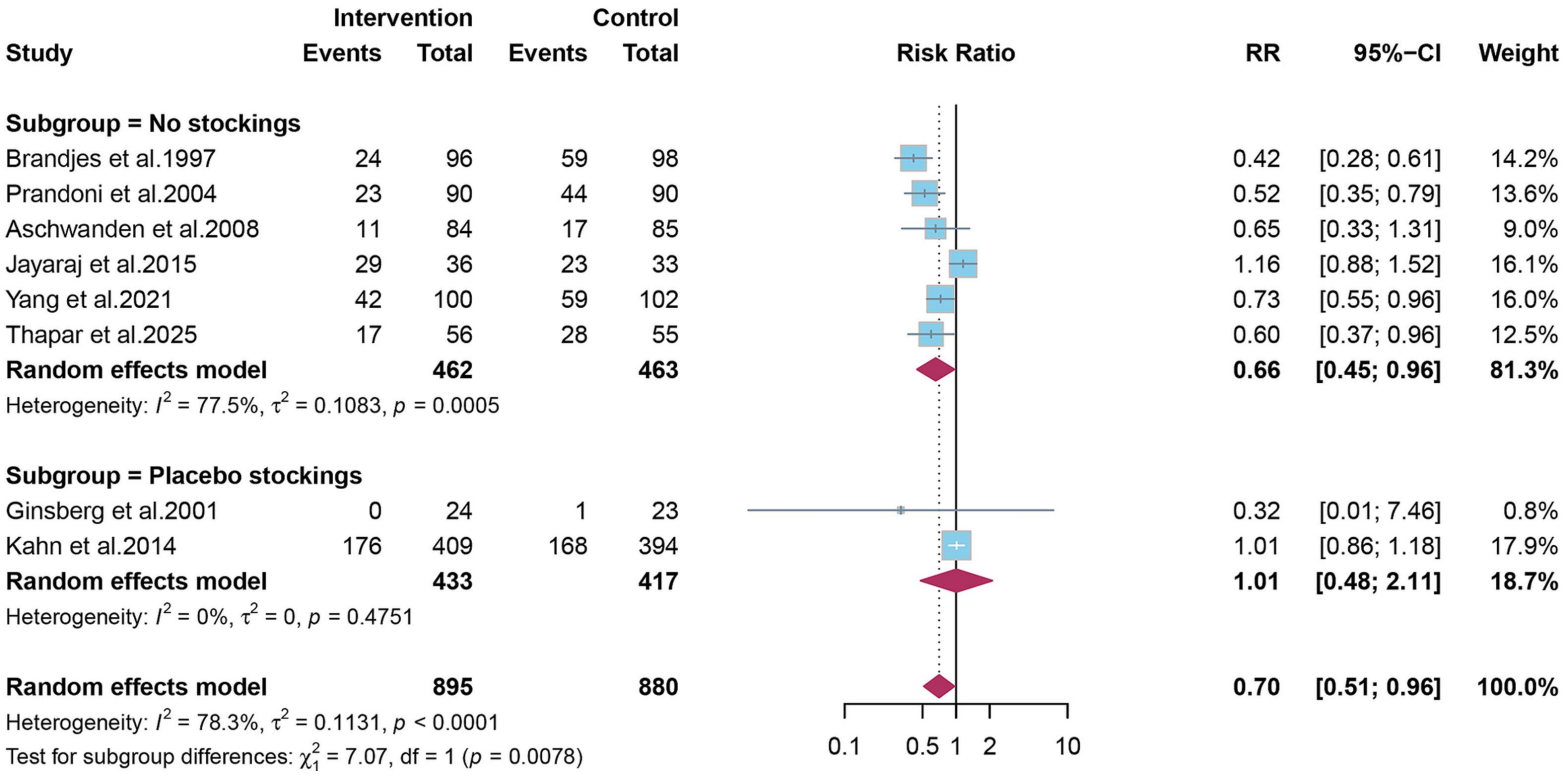
Subgroup analysis on control group intervention.

After conducting subgroup analyses based on PTS severity (mild-to-moderate and severe) and stratified by the control group intervention (placebo vs. no stocking), we found that classification by PTS severity did not show a significant subgroup effect (*p* = 0.5717) (Figure 7). However, stratification by whether the control group used placebo stockings revealed a significant subgroup effect (*p* = 0.0078) (Figure 8).

Meta-regression analysis was performed to assess the potential influence of adherence on the effect of compression stockings. The results indicated a significant association between adherence and treatment effect (estimate = −2.22, SE = 0.781, Z = −2.845, *p* = 0.004), with a 95% confidence interval ranging from −3.753 to −0.691. This suggests that higher adherence to stocking use is associated with a greater reduction in PTS risk.

Leave-one-out sensitivity analyses indicated that the pooled estimates were somewhat sensitive to individual studies; however, all point estimates remained below 1, suggesting that the effect direction consistently favors the intervention. (Supplementary material S3).

Given that fewer than 10 studies were available for each outcome, publication bias was assessed using Begg’s test, which showed no evidence of significant bias (all *p* > 0.05) (Supplementary material S4).

According to the GRADE framework, the certainty of evidence was downgraded due to imprecision and inconsistency. The certainty was rated as very low for severe PTS and low for total PTS, mild-to-moderate PTS, DVT recurrence, and all-cause mortality (Table 2).

**Table 2:** GRADE assessment of outcome.

Outcome and follow-up	No. of participants (No. of studies and type)	Relative effect (95% CI)	Absolute (95% CI)			Certainty of evidence (quality of evidence)	Reasons for downgrading	Plain language summary
			Elastic stockings	Placebo or no stockings	Difference			
Total PTS	1775 (8 RCTs)	0.70 [0.51,0.96]	313	440	127 fewer per 1,000 (212 fewer to 10 fewer)	Low	Imprecision: serious; Inconsistency: serious.	Elastic stockings may decrease total PTS
Mild-to-moderate PTS	1,659 (6 RCTs)	0.69 [0.50,0.93]	287	416	129 fewer per 1,000 (208 fewer to 29 fewer)	Low	Imprecision: serious; Inconsistency: serious.	Elastic stockings may decrease mild-to-moderate PTS
Severe PTS	1,490 (5 RCTs)	0.56 [0.22,1.43]	30	54	24 fewer per 1,000 (42 fewer to 23 more)	Very low	Imprecision: very serious; Inconsistency: serious.	We are very uncertain about the effect of elastic stockings on severe PTS
Recurrence of DVT	1,379 (4 RCTs)	0.90 [0.73, 1.11]	104	115	11 fewer per 1,000 (31 fewer to 13 more)	Low	Imprecision: very serious.	We are very uncertain about the effect of elastic stockings on recurrence of DVT
All-cause mortality	1,595 (6 RCTs)	0.98 [0.68,1.40]	62	63	1 fewer per 1,000 (20 fewer to 25 more)	Low	Imprecision: Very serious.	We are very uncertain about the effect of elastic stockings on all-cause mortality

## Discussion

In this systematic review and meta-analysis, we synthesized evidence from eight randomized controlled trials including 1,775 patients to evaluate the effect of long-term elastic compression stockings (ECS) on post-thrombotic syndrome (PTS) following deep vein thrombosis (DVT). Overall, ECS appeared to reduce the incidence of PTS, primarily driven by a modest effect on mild-to-moderate PTS. However, the effect on severe PTS, recurrent DVT, and all-cause mortality remains highly uncertain, with the certainty of evidence ranging from low to very low.

First, the reduction in mild-to-moderate PTS should be interpreted cautiously. Our analyses indicated that adherence is a key determinant of ECS effectiveness, with higher compliance significantly associated with lower PTS risk. Study design also influenced the observed effects: trials with no-stocking controls demonstrated more pronounced benefit, whereas placebo-controlled trials showed no significant effect. This suggests that open-label studies may overestimate benefit due to subjective assessment, particularly when using scales such as Villalta. Therefore, the modest reduction in mild-to-moderate PTS likely reflects a combination of symptomatic relief, adherence, and trial-specific factors, rather than a definitive preventive effect.

Second, our findings must be interpreted in the context of current clinical guidelines. The 2021 CHEST guideline explicitly recommends against the routine use of ECS for PTS prevention in patients with lower-limb DVT, and the 2020 ASH guideline provides a similar recommendation (39, 40). These recommendations are primarily based on high-quality, placebo-controlled trials, notably the SOX trial (*n* = 806), which found no significant preventive effect of ECS (12). Our meta-analysis aligns with these findings, showing only a modest reduction in mild-to-moderate PTS and no significant effect on severe PTS. Clinically, ECS should not be considered a stand-alone or decisive preventive intervention.

Third, PTS development is influenced by multiple factors beyond compression therapy (41). The quality and intensity of initial DVT management—including the timing, dosage, and monitoring of anticoagulation, early mobilization, thrombus burden, and use of thrombolysis or catheter-based interventions—substantially affect progression to severe PTS (42). In older warfarin-era trials, the absence of data on time in therapeutic range (TTR) further limits interpretability of long-term outcomes (43). Additionally, early venous recanalization and residual venous obstruction are critical determinants, as incomplete recanalization or persistent obstruction increases the risk of severe PTS (44, 45). These observations indicate that ECS should be considered as part of a multifactorial management strategy, rather than a single preventive measure. Variability in treatment intensity, timing, adherence, and monitoring between patients and studies may independently influence the risk and severity of post-thrombotic syndrome (PTS). This clarifies that PTS severity may reflect not only the effect of compression therapy but also differences in the effectiveness of initial DVT management.

Fourth, it is important to distinguish symptom control from disease prevention (41). ECS may provide relief from leg pain, heaviness, or mild swelling, thereby improving quality of life, but current evidence does not support a clear effect on the long-term development of PTS (9). Clinicians and patients should understand that symptom improvement does not necessarily translate into modification of disease progression, highlighting the importance of combining ECS with optimized DVT management and individualized risk assessment (9).

Finally, our findings have implications for future trial design and clinical practice. High-quality, blinded, placebo-controlled trials such as SOX should be given greater weight when interpreting pooled results. Adherence management, patient education, and ease of use remain key determinants of ECS effectiveness in real-world practice. Standardized reporting of adherence, clear specifications of wearing duration and pressure, and rigorous documentation of initial DVT management would enhance interpretability and reduce confounding in future studies.

This study has several limitations. First, the number of included trials was relatively small, with fewer than 10 studies available for each outcome, which limited statistical power and precluded a comprehensive assessment of publication bias. Second, clinical heterogeneity existed across studies, including variations in compression pressure, duration of stocking use, patient characteristics, and diagnostic criteria for PTS (e.g., Villalta, Ginsberg, and CEAP), which may have influenced pooled estimates and reduced comparability. Third, adherence to compression therapy—a key determinant of treatment effectiveness—was inconsistently reported, potentially introducing bias and affecting the observed effects. Fourth, loss to follow-up in some studies further limited the robustness of findings. Fifth, in our study, a uniform minimal important difference (MID) value was applied for evaluating the clinical significance of binary outcomes, based on approaches used in previous evidence syntheses. While this method provided transparency and standardization, it may not be fully appropriate for all outcomes, such as recurrent DVT, mortality, or severe PTS, and could influence judgments regarding imprecision. Sixth, the included studies used different PTS assessment tools, including the Villalta scale, Ginsberg criteria, and CEAP classification, which are not fully interchangeable. Although we used each study’s own severity boundaries, pooled outcomes as relative risks, and applied random-effects models to account for within- and between-study variability, the inability to fully harmonize PTS definitions remains a limitation. Therefore, readers should interpret the pooled estimates with caution, considering the potential impact of this heterogeneity. Finally, despite using the GRADE framework, the certainty of evidence for most outcomes was low to very low due to imprecision and inconsistency. Consequently, conclusions regarding severe PTS, recurrent DVT, and long-term mortality should be interpreted with caution. Although ECS may provide some protection against mild-to-moderate PTS, its clinical application should be guided by patient-specific factors, the quality of initial DVT management, and a multifactorial preventive approach.

## Conclusion

Long-term use of elastic compression stockings may provide modest protection against mild-to-moderate PTS following DVT, but their effect on severe PTS, recurrent DVT, and all-cause mortality remains uncertain. Evidence is of low to very low certainty and is influenced by adherence, control group type, quality of initial DVT management, venous recanalization, and residual obstruction. ECS can be considered an adjunct for symptom relief and quality-of-life improvement, but it should not be used as a stand-alone preventive measure. Clinical decisions should integrate patient-specific risk factors, quality of DVT management, and adherence considerations, emphasizing multifactorial intervention and individualized care rather than reliance on ECS alone.

## Data Availability

The original contributions presented in the study are included in the article/Supplementary material, further inquiries can be directed to the corresponding author.
