# Supplementary material for: Effectiveness of long-term graduated compression stockings after deep vein thrombosis: a GRADE-based meta-analysis of randomized controlled trials
Source: Front Med (Lausanne). 2026 May 21;13:1840243. doi: 10.3389/fmed.2026.1840243 (PMC13233196; doi:10.3389/fmed.2026.1840243)
Supplement: Supplementary file 1 [file Supplementary_file_1.docx]

S1. Search strategy

PubMed

1. “Postthrombotic Syndrome" [Mesh] OR "Postphlebitic Syndrome" [Mesh] OR "Postthrombotic Syndrome"[tiab] OR “Venous Stasis Syndrome”[tiab] OR “Postphlebitic Syndrome”[tiab] OR (postthrombo*[tiab] or postphlebit*[tiab] or post thrombo*[tiab] or “post phlebit*”[tiab])
2. "Stockings, Compression"[Mesh] OR "Compression Stockings"[tiab] OR “Compression Stocking”[tiab] OR “Elastic Stockings”[tiab] OR “Elastic Stocking”[tiab] OR (bandage*[tiab] or stocking*[tiab] or sock*[tiab] or compress*[tiab] or elastic*[tiab] or legging*[tiab] or hose*[tiab] or hosiery*[tiab])
3. rando* [tiab]

Cochrane Library

1. (Postthrombotic Syndrome OR Postphlebitic Syndrome OR Venous Stasis Syndrome OR postthrombo* OR postphlebit* OR post thrombo* OR post phlebit*):ti,ab
2. (Compression Stockings OR Compression Stocking OR Elastic Stockings OR Elastic Stocking OR bandage* OR stocking* OR sock* OR compress* OR elastic* OR legging* OR hose* OR hosiery*):ti,ab
3. (rando*):ti,ab

Embase

1. 'postthrombosis syndrome'/exp

2.((Postthrombotic Syndrome) OR (Postphlebitic Syndrome) OR (Postthrombotic Syndrome) OR (Venous Stasis Syndrome) OR (Postphlebitic Syndrome) OR (postthrombo*) OR (postphlebit*) OR (post thrombo*) OR (post phlebit*)):ti,ab

3. #1 OR #2

4. 'compression stocking'/exp

5. ((Compression Stockings) OR (Compression Stocking) OR (Elastic Stockings) OR (Elastic Stocking) OR (bandage*) OR (stocking*) OR (sock*) OR (compress*) OR (elastic*) OR (legging*) OR (hose*) OR (hosiery*)):ti,ab

6. #4 OR #5

7. (rando*):ti,ab

8. #3 AND #6 AND #7

Web of Science

1. TS=( Postthrombotic Syndrome OR Postphlebitic Syndrome OR Venous Stasis Syndrome OR postthrombo* OR postphlebit* OR post thrombo* OR post phlebit*)
2. TS=( Compression Stockings OR Compression Stocking OR Elastic Stockings OR Elastic Stocking OR bandage* OR stocking* OR sock* OR compress* OR elastic* OR legging* OR hose* OR hosiery*)
3. TS=rando*
4. #1 AND #2 AND #3

S2. Risk of bias

| Study | Domain1 | Domain2 | Domain3 | Domain4 | Domain5 | Total |
| --- | --- | --- | --- | --- | --- | --- |
| Brandjes et al.1997 | Low risk | Low risk | Low risk | Low risk | Low risk | Low risk |
| Ginsberg et al.2001 | Low risk | Low risk | Low risk | Low risk | Low risk | Low risk |
| Prandoni et al.2004 | Low risk | High risk | Low risk | Low risk | Low risk | High risk |
| Aschwanden et al.2008 | Low risk | High risk | Low risk | Low risk | Low risk | High risk |
| Khan et al. 2014 | Low risk | Low risk | Low risk | Low risk | Low risk | Low risk |
| Jayaraj et al.2015 | Low risk | Low risk | High risk | Low risk | Low risk | High risk |
| Yang et al.2021 | Low risk | Low risk | Low risk | Low risk | Low risk | Low risk |
| Thapar et al.2025 | Low risk | High risk | High risk | Low risk | Low risk | High risk |

Domain1: Bias arising from the randomization process

Domain2: Bias due to deviations from intended interventions

Domain3: Bias due to missing outcome data

Domain4: Bias in measurement of the outcome

Domain5: Bias in selection of the reported result

S3. Leave-one-method

Table1. mild-to-moderate PTS

|  | RR 95%-CI | p-value | tau^2 | tau | I^2 |
| --- | --- | --- | --- | --- | --- |
| Omitting Brandjes et al.1997 | 0.77 [0.59; 1.00] | 0.05 | 0.03 | 0.16 | 0.39 |
| Omitting Prandoni et al.2004 | 0.70 [0.47; 1.04] | 0.07 | 0.07 | 0.27 | 0.69 |
| Omitting Aschwanden et al.2008 | 0.69 [0.46; 1.01] | 0.05 | 0.07 | 0.27 | 0.72 |
| Omitting Kahn et al.2014 | 0.62 [0.46; 0.83] | 0.01 | 0.02 | 0.12 | 0.10 |
| Omitting Yang et al.2021 | 0.66 [0.44; 1.00] | 0.05 | 0.08 | 0.29 | 0.72 |
| Omitting Thapar et al.2025 | 0.68 [0.46; 1.02] | 0.06 | 0.08 | 0.28 | 0.72 |
| Random effects model | 0.69 [0.50; 0.93] | 0.03 | 0.06 | 0.24 | 0.66 |

Table2. Severe PTS

|  | RR 95%-CI | p-value | tau^2 | tau | I^2 |
| --- | --- | --- | --- | --- | --- |
| Omitting Brandjes et al.1997 | 0.54 [0.12; 2.42] | 0.28 | 0.61 | 0.78 | 0.56 |
| Omitting Prandoni et al.2004 | 0.65 [0.19; 2.23] | 0.35 | 0.34 | 0.58 | 0.57 |
| Omitting Kahn et al.2014 | 0.41 [0.24; 0.71] | 0.01 | 0.00 | 0.00 | 0.00 |
| Omitting Yang et al.2021 | 0.58 [0.16; 2.07] | 0.27 | 0.40 | 0.63 | 0.65 |
| Omitting Thapar et al.2025 | 0.61 [0.20; 1.84] | 0.25 | 0.33 | 0.57 | 0.62 |
| Random effects model | 0.56 [0.22; 1.43] | 0.16 | 0.35 | 0.59 | 0.56 |

Table3. Recurrence DVT

|  | RR 95%-CI | p-value | tau^2 | tau | I^2 |
| --- | --- | --- | --- | --- | --- |
| Omitting Brandjes et al.1997 | 0.85 [0.73; 1.00] | 0.0476 | 0 | 0 | 0% |
| Omitting Prandoni et al.2004 | 0.90 [0.61; 1.32] | 0.3433 | 0 | 0 | 0% |
| Omitting Kahn et al.2014 | 0.98 [0.68; 1.41] | 0.8453 | 0 | 0 | 0% |
| Omitting Yang et al.2021 | 0.91 [0.65; 1.27] | 0.3445 | 0 | 0 | 0% |
| Random effects model | 0.90 [0.73; 1.11] | 0.21 | 0.00 | 0.00 | 0.00 |

Table4. All-cause mortality

|  | RR 95%-CI | p-value | tau^2 | tau | I^2 |
| --- | --- | --- | --- | --- | --- |
| Omitting Brandjes et al.1997 | 0.90 [0.56; 1.43] | 0.56 | < 0.0001 | 0.00 | 0.00 |
| Omitting Ginsberg et al.2001 | 0.95 [0.69; 1.32] | 0.71 | 0.00 | 0.00 | 0.00 |
| Omitting Prandoni et al.2004 | 1.05 [0.73; 1.52] | 0.71 | < 0.0001 | 0.00 | 0.00 |
| Omitting Aschwanden et al.2008 | 0.97 [0.62; 1.52] | 0.88 | < 0.0001 | 0.00 | 0.00 |
| Omitting Kahn et al.2014 | 0.98 [0.51; 1.90] | 0.94 | 0.02 | 0.15 | 0.00 |
| Omitting Yang et al.2021 | 0.99 [0.65; 1.51] | 0.94 | < 0.0001 | 0.00 | 0.00 |
| Random effects model | 0.98 [0.68; 1.40] | 0.87 | < 0.0001 | 0.00 | 0.00 |

S4. Publication bias by Begger’s test

| Outcome | P values |
| --- | --- |
| mild-to-moderate PTS | 0.57 |
| Severe PTS | NA |
| Recurrence DVT | 1.00 |
| All-cause mortality | 0.57 |

NA, not available

S5. Calculating absolute effect based on baseline risk and relative effect on Total PTS

The risk in the control group (estimated as the median risk in placebo groups) is 50%.

1. Risk with intervention (corresponding risk) = RR x Risk with control

=50%*0.70

=35%

(2) For the upper limit of the RR CI (0.96): • Risk with intervention = 0.96 x50% = 48%

(3) For the lower limit of the RR CI (0.51): • Risk with intervention = 0.51 x 50% = 25.5%
